# Comparison of Predictive Powers for Mortality between Systemic Vascular Resistance Index and Serum Lactate in Children with Persistent Catecholamine-Resistant Shock

**DOI:** 10.1155/2020/1341326

**Published:** 2020-06-19

**Authors:** En-Pei Lee, Sheng-Chih Chu, Shao-Hsuan Hsia, Kuan-Fu Chen, Oi-Wa Chan, Chia-Ying Lin, Ya-Ting Su, Jainn-Jim Lin, Han-Ping Wu

**Affiliations:** ^1^Division of Pediatric Critical Care Medicine, Department of Pediatrics, Chang Gung Memorial Hospital at Linkou, Kweishan, Taoyuan, Taiwan; ^2^College of Medicine, Chang Gung University, Taoyuan, Taiwan; ^3^Department of Pediatrics, Taoyuan Armed Forces General Hospital, Taoyuan, Taiwan; ^4^College of Medicine, National Defense Medical Center, Taipei, Taiwan; ^5^Department of Emergency Medicine, Chang Gung Memorial Hospital at Linkou, Taoyuan, Taiwan; ^6^Department of Emergency Medicine, Chang Gung Memorial Hospital at Keelung, Keelung, Taiwan; ^7^Clinical Informatics and Medical Statistics Research Center, Chang Gung University, Taoyuan, Taiwan; ^8^Community Medicine Research Center, Chang Gung Memorial Hospital Keelung, Keelung, Taiwan; ^9^Department of Pediatric Emergency Medicine, Children's Hospital, China Medical University, Taichung, Taiwan; ^10^Department of Medical Research, Children's Hospital, China Medical University, Taichung, Taiwan; ^11^Department of Medicine, School of Medicine, China Medical University, Taichung, Taiwan

## Abstract

Persistent catecholamine-resistant shock in children causes severe morbidity and mortality. We aimed to analyze the association between hemodynamics and serum lactate at different time points and 28-day mortality in children with persistent catecholamine-resistant shock. *Methods*. Twenty-six children with persistent catecholamine-resistant shock were enrolled, and their hemodynamics were monitored using the pulse index continuous cardiac output. Serial cardiac index (CI), systemic vascular resistant index (SVRI), and vasoactive-inotropic score (VIS) were analyzed for the first 24 hours. Associations between hemodynamics, serum lactate, and 28-day mortality were analyzed. *Results*. The 28-day mortality rate was 53.8%. SVRI and VIS were independent predictors of 28-day mortality. The mortality group had lower serial SVRI and higher VIS than the survival group (*p* < 0.05). Serial SVRI had the highest area under the receiver operating characteristic curve (AUC) for predicting mortality during the first 24 hours of persistent catecholamine-resistant shock. Three important hemodynamic parameters, CI, SVRI and perfusion pressure (MAP-CVP), were significantly correlated with lactate, of which SVRI had the best correlation (*r* = −0.711, *p* < 0.001). According to the AUC, SVRI was a more powerful predictor of mortality than lactate in persistent catecholamine-resistant shock. After 24 hours of treatment for persistent catecholamine-resistant shock, an SVRI lower than 1284 dyn·s·cm^−5^·m^2^ was associated with 28-day mortality. *Conclusions*. SVRI was an early factor associated with mortality in the pediatric patients with persistent catecholamine-resistant shock potentially and had the good correlation with serum lactate. An SVRI more than 1284 dyn·s·cm^−5^·m^2^ during the first 24 hours of persistent catecholamine-resistant shock was associated with favorable outcomes. The result should be used with caution due to the small sample size.

## 1. Introduction

Pediatric severe sepsis remains an important public health issue, with the similar rates of incidence, morbidity, and mortality comparing to critically ill adult populations [1]. Severe sepsis accounted for >8% of all critically ill children and caused more than 4.5 million childhood deaths every year worldwide [1, 2]. In the United States, about one-third of deaths result from severe sepsis in pediatric intensive care units [1]. Furthermore severe sepsis resulted in serious health problems in children, with estimated costs US$ 64280 per patient, causing total annual cost of $4.8 billion in the USA [2]. Persistent catecholamine-resistant shock is the most serious form of sepsis and is defined as hypotension with end-organ dysfunction after fluid resuscitation and vasopressor therapy, with a very high mortality rate of about 60% [3–5].

In practice, clinical hemodynamic and biochemical parameters can be used to diagnose sepsis. The American College of Critical Care Medicine suggests that hemodynamic parameters are important and can be the resuscitation endpoint after adjusting for fluid resuscitation and vasoactive agents [6]. Previous studies have shown that persistent abnormal hemodynamics such as lower cardiac index (CI) and systemic vascular resistant index (SVRI) are associated with increasing mortality in pediatric patients with septic shock [7–10]. In addition, Ceneviva et al. reported that optimal CI and SVRI were associated with a better prognosis in children with persistent catecholamine-resistant shock [11].

With regard to biochemical parameters, elevated serum lactate has been shown to be a powerful predictor of morbidity and mortality in septic patients, and an early serum lactate level higher than 36 mg/dL has been strongly associated with mortality in pediatric patients with sepsis [12]. In addition, both in vivo and in vitro experimental studies have shown that lactic acidosis can damage the cardiovascular system by worsening myocardial and vascular function causing a poor response of vascular tone to vasopressors, although these effects have not been formally established in humans [13–15]. To date, no published paper has demonstrated a relationship between lactate and important hemodynamic parameters such as CI and SVRI in patients with sepsis.

The primary aims of this study were to identify early predictors of mortality in pediatric patients with catecholamine-resistant shock using hemodynamic and biochemical parameters and their optimal cut-off values for predicting mortality and to compare their predictive power. The secondary aim was to analyze the relationships between the hemodynamic parameters and lactic acidosis in these children.

## 2. Methods

### 2.1. Patient Population and Study Design

This was a retrospective cohort study using chart reviews of children aged 1 month to 18 years presenting with shock at the pediatric intensive care unit (PICU) of Chang Gung Children's Hospital between January 1, 2008 and December 31, 2017. The PICU of our hospital is a tertiary ICU with 29 beds located in northern Taiwan. A diagnosis of septic shock was made according to the 2005 International Pediatric Sepsis Consensus Conference [4]. Fluid-refractory septic shock was defined as persistent shock despite up to 60 mL/kg of fluid resuscitation based on Surviving Sepsis Campaign 2012 [6, 10, 16]. Catecholamine-resistant shock was defined as persistent hypotension after fluid replacement and the support of one kind of vasoactive agent [4, 5]. Persistent catecholamine-resistant shock was defined as persistent hypotension after the use of two kinds of vasoactive agents. Patients were included in the study if they were diagnosed with sepsis complicated with persistent catecholamine-resistant shock, and their hemodynamics were monitored via a pulse index continuous cardiac output system (PiCCO, PULSION Medical Systems, Munich, Germany). The following information was recorded: (1) demographic data: age, sex, pediatric risk of mortality (PRISM) score, and site of infection; (2) cardiac characteristics: including initial vasoactive-inotropic score (VIS), heart rate (beats/min), and mean arterial pressure (MAP; mmHg); (3) hemodynamic parameters of the PiCCO system; and (4) outcome: length of stay in the hospital and PICU and 28-day mortality. This study was approved by the Institutional Review Board of Chang Gung Memorial Hospital.

### 2.2. Measurement of PiCCO Parameters and Serum Lactate

The PICCO system was executed within 2 hours of enrollment. The inserted site of central venous and arterial catheter was the internal jugular vein and femoral artery, respectively. Three cold boluses (each bolus: 15 mL 0.9% saline at a temperature < 8°C) were performed for each calibration. The calibration was performed at least every 8 hours, or following an unexpected change of hemodynamics. The continuous parameters of SVRI and CI were recorded and exported to a computer using PiCCO-VoLEF Data Acquisition software (version 6.0; PULSION Medical Systems) combined with a PiCCOPlus device (PC 8100 software version 5.1). The hemodynamic parameters obtained via the PiCCO system included the following: (1) preload parameters: global end-diastolic volume index (GEDVI), intrathoracic blood volume index (ITBVI), and stroke volume variation (SVV); (2) cardiac parameters: cardiac output (CO), cardiac index (CI), and global ejection fraction (GEF); (3) afterload parameter: systemic vascular resistance index (SVRI); and (4) lung parameters: extravascular lung water index (EVLWI) and pulmonary vascular permeability index (PVPI). Serial hemodynamic parameters during the first 24 hours were analyzed. Initial parameters were detected within 1 hour after PiCCO setup. Other data were obtained hourly after the initiation of critical care via the PiCCO system. In addition, the serum lactate level was recorded every 6 hours after the PiCCO system had been implanted.

### 2.3. Statistical Analysis

In the descriptive analysis, data are presented as means ± standard deviations (SDs) or median and interquartile range (IQR, 25th-75th percentile) and number (%). For comparisons of dichotomous variables between groups, the chi-square test or Fisher's exact test was used. Comparisons of continuous variables between the two groups were performed using the Mann–Whitney *U* test. Predicted probabilities of mortality and 95% confidence intervals (CIs) were calculated using a logistic regression model. Receiver operating characteristic (ROC) curves were then used to determine the ideal cut-off values of the hemodynamic parameters for mortality. The characteristics of the cut-off values, including sensitivity, specificity, area under the ROC curve (AUC), positive likelihood ratio (LR^+^), and negative likelihood ratio (LR^−^) were also calculated. Statistical significance was set at *p* < 0.05. All statistical analyses were performed using SPSS software (version 22.0; SPSS Inc., Chicago, IL, USA).

## 3. Results

### 3.1. Demographics of the Children Implanted with the PiCCO Device

During the 10-year study period, 26 children (13 males and 13 females) with persistent catecholamine-resistant shock were monitored using the PiCCO system (Table 1). Of these patients, 14 (53.9%) died within 28 days and 12 (46.1%) survived. There was no significant difference in mean age between the two groups. The most common site of infection in both groups was the blood stream, and microbiologically proven infections were observed in 83.3% of the survival group compared to 78.5% of the mortality group (*p* > 0.05). Gram-negative pathogens were most common in both groups. Dopamine and epinephrine were the first and most commonly used vasoactive-inotropic agents, with no significant difference between the two groups. The initial cardiac characteristics showed higher VIS, lower MAP, and higher serum lactate level in the 28-day mortality group than in the survival group (*p* < 0.05).

### 3.2. Initial and Serial Hemodynamic Parameters and Mortality

**Figure 1:**
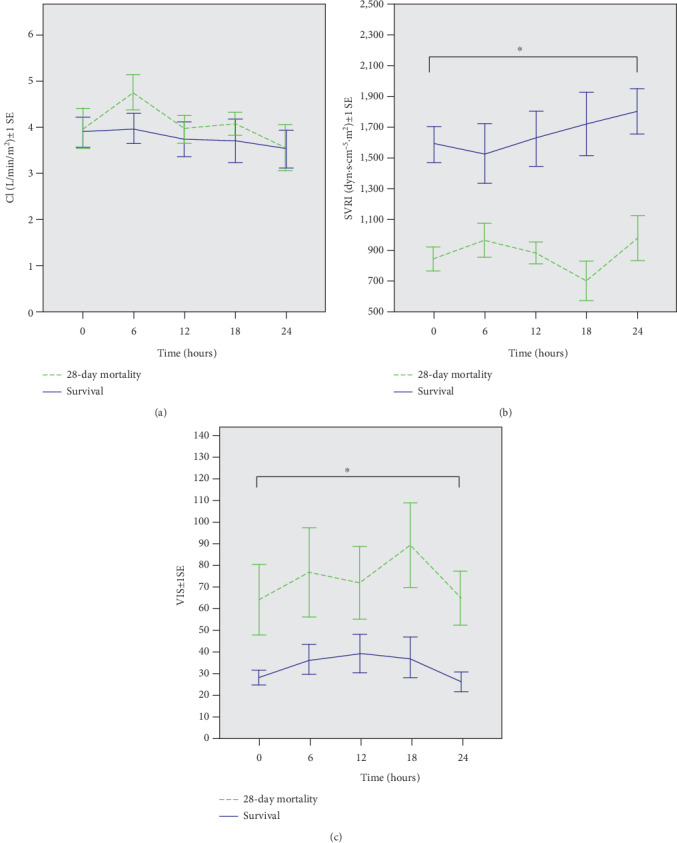
The means of serial cardiac index (CI), systemic vascular resistance index (SVRI), and vasoactive-inotropic score (VIS) after PiCCO setup measured at 6-hour intervals between the survival and 28-day mortality groups. (a) Serial CI. (b) Serial SVRI. (c) Serial VIS. (The I bars indicate standard error of the mean within each time interval.) ^∗^*p* < 0.05.

As shown in Table 1, there were no significant differences in the initial hemodynamic parameters of CO, cardiac contractility, preload, and lung parameters between the two groups. Only SVRI was significantly lower in the 28-day mortality group than the survival group (849 ± 286.8 vs. 1591.2 ± 409.5 dyn·s·cm^−5^·m^2^, *p* < 0.001).  Figure 1 shows the three most important parameters measured at 6-hour intervals after the PiCCO device had been set-up for the first 24 hours. Serial CI values gradually decreased after 24 hours of treatment and was not significantly different between the two groups. However, SVRI gradually increased after 24 hours of treatment and was significantly higher in the survival group. VIS was significantly higher in the mortality group. The results of multivariate logistic regression analysis showed that SVRI and VIS were independent predictors of 28-day mortality and that serial SVRI had the highest AUC for predicting mortality (Figure 1).

### 3.3. Correlations between Lactate and Hemodynamic Parameters

**Figure 2:**
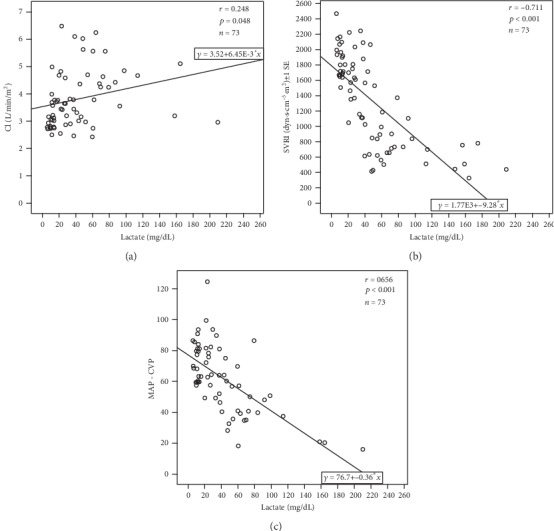
Correlation between lactate and hemodynamics. (a) Cardiac index (CI) and lactate. (b) Systemic vascular resistance index (SVRI) and lactate. (c) Perfusion pressure (=mean artery pressure- (MAP-) central venous pressure (CVP)) and lactate.

We analyzed correlations between all of the hemodynamics measured by the PiCCO system and lactate. There were statistically significant correlations between CI (*r* = 0.248, *p* = 0.048), SVRI (*r* = −0.711, *p* < 0.001), and perfusion pressure (MAP-CVP) (*r* = −0.656, *p* < 0.001) with lactate (Figure 2). Furthermore, SVRI had the best negative correlation with lactate.

### 3.4. Survival Analysis

**Figure 3:**
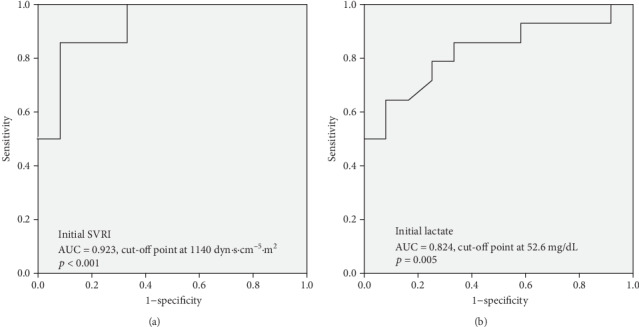
Receiver operating characteristic (ROC) curves and the area under the ROC curves (AUC) and cut-off points of initial SVRI and simultaneous serum lactate for predicting 28-day mortality. Initial SVRI (a, AUC 0.923, cut-off point 1140 dyn·s·cm^−5^·m^2^, *p* < 0.001) was more powerful than simultaneous serum lactate (b, AUC 0.824, cut-off point 52.6 mg/dL, *p* = 0.005) for predicting 28-day mortality.

**Figure 4:**
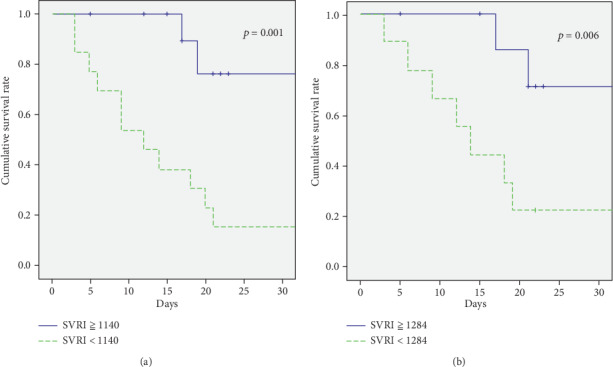
Survival at day 28 related to systemic vascular resistance index (SVRI). The survival rate was (a) only 7.7% if the initial SVRI was less than 1140 dyn·s·cm^−5^·m^2^ and (b) 11.1% if the SVRI was less than 1284 dyn·s·cm^−5^·m^2^ after 24 hours of treatment.

We compared the predictive power of initial SVRI values and serum levels of lactate for 28-day mortality and found that initial SVRI had a larger AUC than lactate (AUC 0.923 and 0.824, respectively) (Figures 3(a) and 3(b)). The best predictive powers for different cut-off values of SVRI in the patients with persistent catecholamine-resistant shock at each 6-hour interval within the first 24 hours are shown in Table 2. The best cut-off values of initial and 24 hours SVRI for predicting mortality were 1140 and 1284 dyn·s·cm^−5^·m^2^, respectively. When sepsis progressed to persistent catecholamine-resistant shock initially, the 28-day survival rate was only 7.7% when the initial SVRI was less than 1140 dyn·s·cm^−5^·m^2^ (*p* = 0.001). After 24 hours of treatment for persistent catecholamine-resistant shock, an SVRI lower than 1284 dyn·s·cm^−5^·m^2^ was associated with poor survival (11.1%), whereas the outcome was favorable (80%, *p* = 0.006) when the SVRI increased to more than 1284 dyn·s·cm^−5^·m^2^ (Figures 4(a) and 4(b)).

## 4. Discussion

The evolution of hemodynamics in septic shock is rapid and unpredictable, especially in persistent catecholamine-resistant shock. Therefore, hemodynamic monitoring is essential for the diagnosis and therapeutic management of pediatric catecholamine-resistant septic shock. In this 10-year retrospective study, we found that SVRI was an early factor associated with mortality in pediatric patients with persistent catecholamine-resistant shock potentially and that it had a good correlation with serum lactate. An SVRI more than 1284 dyn·s·cm^−5^·m^2^ during the first 24 hours in the patients with persistent catecholamine-resistant shock was associated with favorable outcomes. To the best of our knowledge, this is the first study to demonstrate a correlation between hemodynamics and serum lactate in children with persistent catecholamine-resistant shock. The conclusion should be used with caution, because the current study is limited by a small sample size.

Peripheral vasodilatation with hyperdynamic CO is the most common initial phenomenon in septic shock. The major pathophysiology of early sepsis is the injured endothelium resulting in peripheral vasodilatation combined with increasing CO [17]. Injured endothelial cells result in arginine-vasopressin system dysfunction, which results in increased secretions of tumor necrosis factor, prostacyclin, lipopolysaccharide, reactive oxidants, interleukin-1, circulating endothelin, and nitric oxide, which reduce peripheral vascular resistance and vascular reactivity to vasoconstrictors, causing refractory hypotension [18, 19]. Previous adult studies have demonstrated that a low SVRI caused by an impaired endothelium is a predictor of mortality in septic patients [20, 21]; however, the clinical application of SVRI has not been well established in children with septic shock.

According to the American College of Critical Care Medicine-Pediatric Advanced Life Support (ACCM-PALS) algorithm, a CI of 3.3-6.0 L/min/m^2^ indicates better outcomes in children with persistent catecholamine-resistant shock [6, 10, 16]. In our study, we used vasoactive-inotropic agents to achieve the target level of 3.3-6.0 L/min/m^2^, and we found that serial SVRI values were significantly lower in the mortality group compared with the survival group. In addition, in the mortality group, despite a gradual increase in SVRI, the prolonged duration of low SVRI meant a longer period of tissue hypoperfusion, indicating more organ damage [20]. Therefore, lower serial SVRI values can be used as an earlier marker of disease severity and insufficient resuscitation. Titrating vasoactive-inotropic agents to increase the SVRI should be performed immediately in children with persistent catecholamine-resistant shock and may potentially be used to guide resuscitation.

Measurements of serum lactate during resuscitation from septic shock are highly recommended in sepsis guidelines [22]. High blood lactate level at admission is associated with mortality in septic and other critically ill children [23, 24]. Serial serum lactate may provide a prognostic tool during resuscitation and a normalized lactate within 4 hours is associated with better outcomes in pediatric sepsis [25]. Tissue hypoperfusion and mitochondria dysfunction are the main course of hyperlactatemia in sepsis. Severe lactic acidosis can also worsen vascular consequences and vasodilatation, so that peripheral vasodilatation and hyperlactatemia affect each other. This may explain the high correlation between lactate and SVRI. However, there is currently no single and effective treatment to reverse hyperlactatemia directly. The use of inotropic agents to elevate CO to improve tissue oxygen delivery for treating hyperlactatemia and low central venous oxygen saturation is a widely accepted therapeutic strategy [26]. In the current study, we demonstrated a positive correlation between lactate and CI (*r* = 0.248, *p* = 0.048) in children with persistent catecholamine-resistant shock, and an even better correlation between lactate and SVRI (*r* = −0.711, *p* < 0.001), which is comparable with results of animal studies [27]. Therefore, when a septic child initially presents with a lower SVRI and hyperlactatemia, vasopressors such as epinephrine or norepinephrine instead of inotropic agents may be administered as the initial resuscitation agents to increase SVRI and decrease “functional shunting.” Further studies comparing different vasoactive agents with different hemodynamic status and lactate clearance are warranted.

The current study used dopamine as the first-line vasoactive-inotropic agents before 2018 based on the 2008 Surviving Sepsis Campaign International Guidelines [28]. In recent years, two randomized control trials demonstrated that epinephrine may be better than dopamine as the first-line vasoactive-inotropic agents in treating pediatric septic shock [29, 30]. But these two studies did not analyze the pathophysiology to explain why epinephrine was better than dopamine in pediatric septic shock. The 2020 Surviving Sepsis Campaign International Guidelines reported that they were unable to recommend a specific first-line vasoactive-inotropic agent for pediatric septic shock. The guidelines only suggested that using epinephrine or norepinephrine, rather than dopamine can be the first-line vasoactive-inotropic agents in pediatric septic shock (weak recommendation, low quality of evidence) [31]. The current study demonstrated that SVRI may be an early and more important parameter than CI for predicting outcomes in pediatric septic shock. Therefore, the *α*-adrenergic agonists such as epinephrine or norepinephrine may be better than dopamine potentially to increase SVRI based on the pathophysiology. Future prospective studies are warranted to analyze the change of hemodynamics and other important parameters on outcomes under different vasoactive-inotropic agents.

This study has several limitations. First, the sample size was small and retrospectively conducted at a single center, and therefore, there were risks of missing data and information bias. However, similar results have been reported in adult and animal studies. Second, the study was conducted over a relatively long period (10 years) during which the therapeutic strategy may have evolved, such as the choice of parameters for resuscitation end-points or the choice of antibiotics, which may have impacted the outcomes. However, the chief of our PICU has implemented early-goal directed therapy since 2001 and administered last-line antibiotics for children with septic shock.

## 5. Conclusions

The current study with very few patients demonstrated that SVRI was an early factor associated with mortality in pediatric patients with persistent catecholamine-resistant shock potential and had a good correlation with serum lactate. A lower SVRI was correlated with hyperlactatemia and was an early risk factor predicting mortality. An elevated SVRI during the first 24 hours of resuscitation in persistent catecholamine-resistant shock was associated with favorable outcomes, and its high correlation with serum lactate may have resulted in the decrease in serum lactate level. Future studies should include more patients to avoid the risk of information bias caused by a small sample size.

## Figures and Tables

**Table 1:** Demographics and initial hemodynamic parameters between the survivors and 28-day mortality group.

Variables	Survival (*n* = 12)	28-day mortality (*n* = 14)	*p* value
Age mean (SD), years	12 (4.7)	12.4 (3.3)	0.877
Sex (male), *n* (%)	5 (41.6)	8 (57.1)	
PRISM, median (IQR)	8.8 (2-19)	11.1 (7-13)	0.188
Site of infection, *n* (%)			0.39
Central nervous system	0	3 (21.4)	
Blood stream	5 (41.6)	6 (42.8)	
Respiratory	4 (33.3)	3 (21.4)	
Urologic	1 (8.3)	0	
Abdominal	1 (8.3)	0	
Others	1 (8.3)	2 (14.2)	
Culture positive, *n* (%)	10 (83.3)	11 (78.5)	0.843
Pathogen, *n* (%)			0.862
Gram positive	4 (33.3)	4 (28.5)	
Gram negative	5 (41.6)	5 (35.7)	
Fungus	1 (8.3)	1 (7.1)	
Virus	0 (0)	1 (7.1)	
Unknown	2 (16.6)	3 (21.4)	
Used vasoactive-inotropic agents, *n* (%)			
Dopamine	11 (91.6)	14 (100)	0.921
Epinephrine	9 (75)	13 (92.8)	0.473
Norepinephrine	4 (33.3)	7 (50)	0.642
Dobutamine	1 (8.3)	7 (50)	0.068
Milrinone	9 (75)	8 (57.1)	0.597
Vasopressin	0	1 (7.1)	0.924
Outcomes (mean ± SD)			
ICU stay (days)	25.9 (18.7)	13.8 (9.4)	0.031^∗^
Length of stay (days)	39.1 (30.1)	21.3 (15.7)	0.099
Cardiac characteristics before PiCCO implantation (mean ± SD)			
Vasoactive-inotropic scores	30.7 (13.9)	65.6 (64.9)	0.027^∗^
Heart rate (beats/min)	118 (32)	143 (28)	0.092
Mean arterial pressure (mmHg)	82.5 (16.7)	53.4 (13.8)	<0.001^∗^
Lactate (mg/dL)	32.3 (14)	74.2 (46.9)	0.005^∗^
Initial hemodynamic parameters (mean ± SD)			
Cardiac output			
CO (L/min)	3.9 (1.52)	4.5 (1.5)	0.273
Cardiac contractility			
CI (L/min/m^2^)	3.7 (1.1)	3.9 (1)	0.603
GEF (%)	32.3 (11.1)	27.2 (8.7)	0.356
CFI (I/min)	9.2 (3.1)	9.1 (3.2)	1
Preload parameters			
GEDVI (mL/m^2^)	431.7 (161.7)	447.1 (150.9)	0.823
ITBVI (mL/m^2^)	539.1 (202.2)	558.6 (188.8)	0.823
SVV (%)	10.3 (3.4)	17.2 (6.9)	0.006^∗^
Afterload parameters			
SVRI (dyn·s·cm^−5^·m^2^)	1591.2 (409.5)	849 (286.8)	<0.001^∗^
Lung parameters			
EVLWI (mL/m^2^)	10.6 (4.4)	12.2 (6.4)	0.507
PVPI	2.9 (0.9)	3.3 (1)	0.326

ICU: intensive care unit; CO: cardiac output; CI: cardiac index; GEF: global ejection fraction; CFI: cardiac function index; GEDVI: global end-diastolic volume index; ITBVI: intrathoracic blood volume index; SVV: stroke volume variation; SVRI: systemic vascular resistance index; EVLWI: extravascular lung water index; PVPI: pulmonary vascular permeability index. ^∗^Statistical significance was set at *p* < 0.05.

**Table 2:** Best predictive power for different cut-off points of systemic vascular resistance index in persistent catecholamine-resistant shock at each 6-hour interval within the first 24 hours.

Time (hours)	SVRI (dyn·s·cm^−5^·m^2^)	Sensitivity	Specificity	LR^+^	LR^−^	Youden index
0	1140	0.8	0.89	7.2	0.23	0.7
6	1011	0.7	0.91	7.7	0.33	0.6
12	977	0.8	0.9	8	0.22	0.7
18	987	0.75	0.9	7.5	0.28	0.7
24	1284	0.75	0.88	6.8	0.28	0.6

SVRI: systemic vascular resistance index; LR^+^: positive likelihood ratio; LR^−^: negative likelihood ratio.

## Data Availability

No data were used to support this study.
